# Integrative Medicine Treatment in Times of Pandemic Coronavirus Disease?

**DOI:** 10.1089/acu.2020.1441

**Published:** 2021-02-16

**Authors:** Juan Yang, Dietlind L. Wahner-Roedler, Tony Y. Chon, Brent A. Bauer

**Affiliations:** Division of General Internal Medicine, Mayo Clinic, Rochester, MN, USA.

**Keywords:** coronavirus, COVID-19, integrative medicine, acupuncture, Chinese medicine, remote medical service

## Abstract

Coronavirus disease (COVID-19) has expanded around the world, resulting in a pandemic with high morbidity and mortality. To date, no specific treatment or vaccine is available to treat or prevent this sudden and potentially deadly disease. Economic restructuring brings opportunities and challenges to integrative medicine treatment. In such complex situations, integrative medicine treatments are to be provided cautiously, and the shift from in-person visits to remote medical services might play an important role in how such services are delivered.

A novel coronavirus disease affecting the respiratory tract of patients and potentially resulting in critical pneumonia was originally reported in *Wuhan, Hubei* province, China in December 2019, and named as coronavirus disease 2019 (COVID-19) by the World Health Organization (WHO) officially on February 11, 2020.^1^ It is another pathogenic viral infection caused by a novel coronavirus, labeled as severe acute respiratory syndrome coronavirus 2 (SARS-CoV-2) transmitted across the animal–human interface. Studies have indicated that its pathogens spread through populations by transmission through respiratory droplets, contact, and natural aerosols.^2^ Fever, tiredness, and dry cough are the most common signs and symptoms of patients with COVID-19, 25.9% of them will develop serious pneumonia requiring intensive care unit (ICU) admission and 20.1% may develop acute respiratory distress syndrome.^3^ Due to alarming levels of spread, and severity, the coronavirus outbreak has been declared as a global pandemic by the WHO on March 11, 2020.^4^ As of June 3, 2020, the virus has spread to 216 countries, areas, and territories, and 6,272,098 confirmed cases and 379,044 confirmed deaths have been reported to the WHO.^5^

Patients with COVID-19 have been found to have higher transmissibility and greater pandemic risk than SARS-CoV.^6^ Given previous experience of management of Middle East Respiratory Syndrome Coronavirus and SARS-CoV, the WHO released infection control interventions recommendations to decrease the general risk of pathogen infection or transmission including wearing facial masks, regular hand cleaning, 6 feet social distance, and stay home policy. Furthermore, health care facilities should improve infection prevention and control practices in clinics and hospitals.^3^

At present, except for supportive care, for example, oxygen therapy, mechanical ventilation, and fluid management, there are no clear or convincing evidence-based interventions known to be of benefit for COVID-19 patients.^7^ Remdesivir is only mentioned as 1 investigational therapy through either compassionate drug use or ongoing clinical trial.^8^ Currently, diagnosis and treatment protocols are developed based on patients from China and other countries around the world to better define this pandemic. Chinese National Health Commission & State Administration of Traditional Chinese Medicine has published the seventh-version Guideline of “The Chinese Clinical Guidance for COVID-19 Pneumonia Diagnosis and Treatment,” which is widely used in clinical practice in China.^9^ The U.S. Centers for Disease Control and Prevention (CDC) published Interim Guidance on Management of COVID-19 to implement aggressive measures to stop the virus spreading in the United States.^10^ Since at the present time there is no vaccine available, the best way to prevent the spread of COVID-19 infection is to avoid being exposed to the virus. Sufficient medical quarantine to reduce exposure and limit transmission to others is a critical first step of COVID-19 prevention and control. Suspected patients need to be quarantined, confirmed patients are transferred to the same general ward, and severe cases should be admitted to the ICU as soon as possible.

Increasing social isolation and loneliness due to widespread outbreak of COVID-19 is inevitable strongly associated with public panic and adverse mental health consequences of psychologic distress and symptoms of mental illness such as anxiety, sadness, stress, depression, self-harm, and even suicide attempts, especially among vulnerable populations and health care workers.^11^

Most COVID-19 patients initially suffer from fever, cough, dyspnea, anxiety, stress, and other symptoms including musculoskeletal pain, nausea, and diarrhea for which patients may first seek help using integrative medicine treatments. These can include diverse therapies such as herbal therapy, acupuncture, massage, mind–body practice, and others. Many of these modalities require face-to-face contact and, therefore, the outbreak of COVID-19 has also posed a great challenge to the clinical practice of integrative medicine therapies.^12^

## Herbal Therapy

Previous studies have shown that Chinese herbal remedy might play a crucial role in the prevention and treatment of epidemic diseases such as SARS-CoV and influenza A virus subtype H1N1 (A/H1N1) with a higher recovery rate and lower medical cost in China.^13,14^ Fourteen patients with influenza A/H1N1 flu were reportedly cured with integrative treatment of Chinese herbal remedies in Beijing Ditan Hospital and Chengdu Infectious Diseases Hospital, China.^13^ Approximately 40%–60% of hospitalized SARS patients received an integrated approach of modern and Chinese herbs. Positive effects of Chinese herbal therapy as an adjuvant showed improvement of fever, chest infection, less steroids consumption, and immunologic boosters.^14^ Chinese National Health Commission & State Administration of Traditional Chinese Medicine released guidelines regarding systemic treatment with Chinese herbal remedies for COVID-19 patients in different stages. For example, in the seventh editions of Guidelines of Traditional Chinese Medicine treatment, Chinese herbal remedy *Huoxiang Zhengqi* capsule is advised for fatigue and gastrointestinal discomfort; *Jinhua Qinggan* granules, *Shufeng Jiedu* capsules, *Lianhua Qingwen* capsules, and *Fangfeng Tongsheng* pills for fatigue and fever of suspected patients; lung cleansing and detoxifying decoction and other herbal decoctions are recommended to confirmed cases; *Xiyanping* and other Chinese medicine injections are applied on severe and critical cases; and it seems that Chinese herbs may have positive effects on major symptoms such as fever, cough, and promote recovery. Further laboratory research and clinical trials with regard to the efficacy and safety of wide application of these herbal remedies on COVID-19 patients are urgently needed^15–17^ (Table 1).

**Table 1.** Chinese Medicine Remedies Recommended for COVID-19

Populations	Population	Syndrome	Symptoms and signs	Chinese Medicine remedies	Usage	Injections
Medical observation period	—	—	Fatigue with gastrointestinal discomfort	*Huoxiang Zhengqi* capsule.	Pills, liquid, or oral solution	—
	—	—	Fatigue with fever	*Jinhua Qinggan* granules, *Lianhua Qingwen* capsules (granules), *Shufeng Jiedu* capsules (granules), *Fangfeng Tongsheng* pills (granules)	Pills, granules	—
Clinical treatment period (confirmed patients)	Mild, moderate, and severe patients and partially critically ill patients.	—	—	*Qingfei Paidu* decoction (lung cleansing and detoxifying decoction) *Ma Huang* 9 g, *Zhi Gan Cao* 6 g, *Xing Ren* 9 g, *Sheng Shi Gao* (decocted first) 15–30 g, *Gui Zhi* 9 g, *Ze Xie* 9 g, *Zhu Ling* 9 g, *Bai Zhu* 9 g, *Fu Ling* 15 g, *Chai Hu* 16 g, *Huang Qin* 6 g, *Jiang Ban Xia* 9 g, *Sheng Jiang* 9 g, *Zi Wan* 9 g, *Kuan Dong Hua* 9 g, *She Gan* 9 g, *Xi Xin* 6 g, *Shan Yao* 12 g, *Zhi Shi* 6 g, *Chen Pi* 6 g, *Huo Xiang* 9 g.	One dose daily, decoction, take warm 2 times (40 minutes after meal in the morning and evening), and 3 doses a course.	—
	Mild cases	Cold damp constraint in the lung pattern	Fever, fatigue, generalized body aches, cough, expectoration, chest tightness, and labored breathing, poor appetite, nausea, vomiting and sticky stool, pale enlarged tongue with tooth marks or light red tongue and coating which is white, thick, curd-like, and greasy or white and greasy, and soggy of slippery pulse.	*Sheng Ma Huang* 6 g, *Sheng Shi Gao* 15 g, *Xing Ren* 9 g, *Qiang Huo* 15 g, *Ting Li Zi* 15 g, *Guan Zhong* 9 g, *Di Long* 15 g, *Xu Chang Qing* 15 g, *Huo Xiang* 15 g, *Pei Lan* 9 g, *Cang Zhu* 15 g, *Yun Ling* 45 g, *Sheng Bai Zhu* 30 g, *Jiao San Xian (Jiao Shan Zha*, *Jiao Shen Qu*, and *Jiao Mai Ya*) 9 g each, *Hou Po* 15 g, *Jiao Bing Lang* 9 g, *Wei Cao Guo* 9 g, *Sheng Jiang* 15 g.	One dose daily, 600 mL after decocting, divide into 3 times, equally in the morning, afternoon and evening, take before meal.	—
		Damp heat accumulation in the lung pattern	Low-grade fever or absence of fever, slight aversion to cold, fatigue, heavy sensation in the head and body, muscle soreness, dry cough with little sputum, sore throat, thirst without desire to drink, or accompanied with chest tightness and epigastric fullness, absence of sweating or disturbed hidrosis, or vomiting with anorexia, loose stool or sticky stool. The tongue is light red and coating is white, thick, and greasy or thin and yellow. The pulse is slippery and rapid or soggy.	*Bing Lang* 10 g, *Cao Guo* 10 g, *Hou Po* 10 g, *Zhi Mu* 10 g, *Huang Qin* 10 g, *Chai Hu* 10 g, *Chi Shao* 10 g, *Lian Qiao* 15 g, *Qing Hao* (added later) 10 g, *Cang Zhu* 10 g, *Da Qjng Ye* 10 g, *Sheng Gan Cao* 5 g.	One dose daily, 400 mL after decocting, divide into 2, taken half in the morning and half in the evening.	—
	Moderate cases	Damp toxin constraint in the lung pattern	Fever, cough with little sputum or yellow sputum, chest tightness and shortness of breath, abdominal distension, and constipation with difficult defecation. The tongue body is dark red, and tongue shape is enlarged. The coating is yellow greasy or yellow dry. The pulse is slippery and rapid or wiry and slippery.	*Sheng Ma Huang* 6 g, *Ku Xing Ren* 15 g, *Sheng Shi Gao* 30 g, *Sheng Yi Yi Ren* 30 g, *Mao Cang Zhu* 10 g, *Guang Huo Xiang* 15 g, *Qing Hao Cao* 12 g, *Hu Zhang* 20 g, *Ma Bian Cao* 30 g, *Gan Lu Gen* 30 g, *Ting Li Zi* 15 g, *Hua Ju Hong* 15 g, *Sheng Gan Cao* 10 g.	One dose daily, 400 mL after decocting, and equally divide into 2, taken in the morning and evening.	—
		Cold damp obstructing the lung pattern	Low-grade fever, unsurfaced fever or no fever, dry cough with little sputum, lassitude, and fatigue, chest tightness, stomach discomfort, or nausea, and loose stool. The tongue is pale or light red and coating is white or white greasy. The pulse is soggy.	*Cang Zhu* 15 g, *Chen Pi* 10 g, *Hou Po* 10 g, *Huo Xiang* (10 g, *Cao Guo* 6 g, *ShengMa Huang* 6 g, *Qiang Huo* 10 g, *Sheng Jiang* 10 g, *Bing Lang* 10 g.	One dose daily, 400 mL after decocting, and equally divide into 2, taken in the morning and evening.	—
	Severe cases	Epidemic toxin blocking the lung pattern	Fever with red face, cough with little yellow and sticky sputum, or blood-stained sputum, chest tightness and short of breath, lassitude, dryness, bitterness and stickiness in the mouth, nausea, and loss of appetite, difficult defecation, and scanty dark urine. The tongue is red with yellow greasy coating. The pulse is slippery and rapid.	Huashi Baidu Formula *Sheng Ma Huang* 6 g, *Xing Ren* 9 g, *Sheng Shi Gao* 15 g, *Gan Cao* 3 g, *Huo Xiang* (added later) 10 g, *Hou Po* 10 g, *Cang Zhu* 15 g, *Cao Guo* 10 g, *Fa Ban Xia* 9 g, *Fu Ling* 15 g, *Sheng Da Huang* (added later) 5 g, *Sheng Huang Qi* 10 g, *Ting Li Zi* 10 g, *Chi Shao* 10 g.	One to 2 doses daily, decoction, 100–200 mL each time, 2–4 times per day, oral administration or nasal feeding.	—
		Blazing of both Qi and ying pattern	High fever with polydipsia, tachypnea and shortness of breath, delirium and unconsciousness, blurred vision or accompanied with macules and papules, or hematemesis, epistaxis or convulsion of the 4 limbs. The tongue is crimson with little or no coating. The pulse is deep, thready and rapid, or floating, large and rapid pulse.	*Sheng Shi Gao* (decocted first) 30–60 g, *Zhi Mu* 30 g, *Sheng Di* 30–60 g, *Shui Niu Jiao* (decocted first) 30 g, *Chi Shao* 30 g, *Xuan Shen* 30 g, *Lian Qiao* 15 g, *Dan Pi* 15 g, *Huang Lian* 6 g, *Zhu Ye* 12 g, *Ting Li Zi* 15 g, *Sheng Gan Cao* 6 g.	One dose daily, decoction, *Shi Gao* and *Shui Niu Jiao* should be decocted first, 100–200 mL each time, 2–4 times per day, oral administration or nasal feeding.	*Xiyanping* injection, *Xuebijing* injection, *Reduning* injection, *Tanreqing* injection, *Xingnaojing* injection.
	Critical cases	Internal blockage and external desertion pattern	Dyspnea, panting on exertion or mechanical ventilation required, accompanied with unconsciousness and dysphoria, sweating, cold extremities. The tongue is dark and purple with thick greasy or dry coating. The pulse is floating and large without root.	Take *Su He Xiang Wan* or *Angong Niuhuang Wan* with the following decoction composed of *Ren Shen* 15 g, *Hei Shun Pian* (decocted first) 10 g, *Shan Zhu Yu* 15 g.	Recommended usage of Traditional Chinese Medicine injections for severe and critical cases.	*Xuebijing* injection, *Reduning* injection, *Tanreqing* injection, *Xingnaojing* injection, *Shenfu* injection, *Shengmai* injection, *Shenmai* injection.
	Convalescent period	Lung spleen Qi deficiency pattern	Shortness of breath, lassitude and fatigue, poor appetite with nausea and vomiting, abdominal fullness, a sense of incomplete evacuation, and sticky loose stool. The tongue is pale and enlarged with white greasy coating.	*Fa Ban Xia* 9 g, *Chen Pi* 10 g, *Dang Shen* 15 g, *Zhi Huang Qi* 30 g, *Chao Bai Zhu* 10 g, *Fu Ling* 15 g, *Huo Xiang* 10 g, *Sha Ren* (added later) 6 g, *Gan Cao* 6 g.	One dose per day, 400 mL after decocting, and equally divide into 2 and taken in the morning and evening.	—
		Qi and Yin deficiency syndrome	Fatigue, shortness of breath, dry mouth, thirst, heart palpitation, profuse sweating, poor appetite, low-grade fever or no fever, dry cough with little sputum. The tongue is dry tongue with scanty fluid. The pulse is thready or weak and forceless.	*Nan Sha Shen* 10 g, *Bei Sha Shen* 10 g, *Mai Dong* 15 g, *Xi Yang Shen* 6 g, *Wu Wei Zi* 6 g, *Sheng Shi Gao* 15 g, *Dan Zhu Ye* 10 g, *Sang Ye* (Mori Folium) 10 g, Lu Gen 15 g, *Dan Shen* 15 g, *Sheng Gan Cao* 6 g.	One dose per day, 400 mL after decocting, and equally divides into 2 and taken in the morning and evening.	—

## Mind–Body Therapies

Psychologic factors are an essential component in the success of public health strategies used for the management of epidemics and pandemics. Studies indicate that subsyndromal psychiatric disorders such as anxiety, distress, and fear are a common response to the COVID-19 pandemic, particularly in the unemployed or vulnerable population, which potentially impact on the ongoing efforts of public communication, hygiene practices, social distancing, vaccination, and antiviral therapy. People with mental anxiety may engage in a variety of maladaptive safety behaviors, which includes compulsive hand washing, panic buying, and social isolation.^18,19^ Mind–body therapies could guide patients to nonpharmacologic approaches to manage their emotions and discomfort. Being simple and useful home-based workouts, Ayurveda, Yoga, *Tai chi*, or meditation have already played a major role in the prevention and postrecovery management of COVID-19.^20,21^ Remote instructions could be offered by health care providers even under a stay-at-home order. And the efficacy and safety of these modalities used in the treatment of emerging coronavirus infections will need to be evaluated in future studies.

### Acupuncture

China Association of Acupuncture-Moxibustion published “Guidance for acupuncture and moxibustion interventions on COVID-19 (Second edition),” which highlights the beneficial effects of Traditional Chinese Medicine interventions including acupuncture and other related therapies such as moxibustion, *Tuina*, traditional physical exercise, and foot bath fumigation. Acupuncture with different acupoints combination for suspected, confirmed, and recovery of COVID-19 patients is recommended separately.^22^ Acupuncture-related therapies such as bloodletting of auricular and hand points for confirmed patients with repeated fever and moxibustion exerted for 10 to 15 minutes at each point for patients in recovery stage as well as other interventions such as scraping, point injection, *Tuina* and others are recommended. Self-interventions are advised to apply at home under the instruction of a physician.^23^ An exploratory study focused on acupuncture treatment in COVID-19 patients reported that Dr. *Zhou*, a critical care medical expert who supported *Wuhan Leishenshan* hospital, applied acupuncture on COVID-19 confirmed patients for shortness of breath, cough, dizziness, insomnia, restlessness, palpitations, diarrhea, and vomiting. In addition, Dr. *Liu*, a Traditional Chinese Medicine expert who also treated COVID-19–infected patients in *Wuhan* with acupuncture, summarized that acupuncture could have positive effects in improving chest congestion, shortness of breath, abdominal discomfort, itchy throat, cough, dizziness, pain, and sweating.^24^

Acupuncture manipulated by licensed acupuncturists involves the insertion of metal needles and heat or cold stimulation into the precise acupoints on human bodies. Acupuncturists are at high risk of getting infected when they closely examine the patient and do acupuncture manipulations; personal protective equipment (PPE) is needed if they operate these treatments; however, a critical shortage of PPE has already posed a major risk of COVID-19 being spread^25^; and protecting the medical workforce is a critical challenge. During these pandemic outbreaks, severe health care provider infections and deaths have already been reported, making the staff vulnerable to significant psychosocial stress.^26,27^

Moreover, it is very inconvenient to do the manipulations wearing triple layer protective gloves either. During the treatment, if medical provider's hand hygiene, PPE, or other infection prevention and control measures are not in place, they are at great risk of infection and possibly become the virus carriers to the other patients, family members, and the community.

Possibly due to the mentioned limitations, during an acute COVID-19 outbreak, acupuncture-related in-person therapies should be cautiously performed since the high risk of possible virus spreading is much greater than any possible benefit. Possibly due to the mentioned limitations, related therapies, acupuncture-related therapies, which require in-person visits were not included in the diagnosis and treatment protocols as one of the first-line treatment options for COVID-19 published either by Chinese National Health Commission & State Administration of Traditional Chinese Medicine or by the U.S. CDC.

Timely treatment and prevention of COVID-19 are paramount for public health and the well-being, whereas the economic impact on the society is another issue we should think about the fact that economic recession itself has a negative effect on health. To prevent the pandemic spread, expand health capacity to care, and to conserve adequate medical staff and supplies for COVID-19 patients, especially PPE, nonemergent, elective medical services, and treatment are limited, many economic policy tools have also been used for a response to the support of social distancing and hygiene. Therefore, in the United States, integrative medicine as a supplementary of conventional medicine is suspended or shut down in response to CDC guidelines, which resulted in an urgent shift from the traditional in-person service model to remote health care.

The continuing COVID-19 pandemic almost affects economic activities in every country on this planet. The global economy is in the deepest contraction since the great depression, a reduction in economic activity reduces the circulation of money, which results in a heavy hit to the middle-class people, salaried people, organized sector, etc.^28^ Unemployment could influence both the physical and mental health, which might aggravate the negative consequences of the pandemic in a vicious circle. It is a great challenge to reopen some economies around the world. At present, in some areas with a low, or relatively low and stable incidence of COVID-19, medical facilities have allowed the flexibility to provide care for patients needing nonemergent non-COVID-19 health care.

Economic recovery under COVID-19 is definitely a double-edged sword, by reopening the economy there is a potential risking of a second wave. Economic restructuring brings opportunities and challenges to integrative medicine treatments. In such a complex situation, integrated medicine treatments need to be cautiously provided with the premise of safety. The health care providers should weigh their own risk and comfort level when deciding whether to continue providing in-person integrative medicine services. No in-person visit is risk free, even if both patient and practitioner appear well. To prevent the spread of COVID-19, in-person integrative medicine appointment should be limited to patients with a clear and documentable urgent medical need. Further suggested safety recommendations during this pandemic include: prescreening each patient by phone before the consultation; staggering appointments so that patients do not overlap; adequate disinfection of any surfaces that may have been contacted, removing or recycling any nonessential items which could be a vector for virus transmission in the healthcare setting. With the adoption of telehealth medical care can safely be provided to patients in appropriate situations. The shift from in-person appointments to remote medical services might play a new important role in the future.

## Conclusion

The global pandemic of COVID-19 has become a public health emergency to the general public and health care providers. Our understanding of this sudden and lethal virus is still very limited. Safe effective antiviral medication and vaccine are still not available, the only thing we can do at this point is aggressively implement appropriate infection prevention and control measures to curb the spread of this virus transmission. Economic restructuring brings opportunities and challenges to integrative medicine treatment. Integrative medicine treatments are to be provided cautiously, and the shift from in-person visits to remote medical services might play a new important role in the coming medical services.
